# Potential Molecular Biomarkers for Predicting and Monitoring Complications in Type 2 Diabetes Mellitus

**DOI:** 10.3390/molecules30224448

**Published:** 2025-11-18

**Authors:** Zia Shariat-Madar, Fakhri Mahdi

**Affiliations:** Division of Pharmacology, School of Pharmacy, University of Mississippi, Oxford, MS 38677, USA; madar@olemiss.edu

**Keywords:** antihyperglycemic agents, microvascular disease, macrovascular disease, predictive biomarkers of T2DM, diabetes complications, neovasculogenesis, atherosclerosis

## Abstract

According to the National Center for Health Statistics (NCHS) of the Centers for Disease Control and Prevention (CDC), type 2 diabetes mellitus (T2DM) remains a major public health issue with a considerable impact on human life, affecting over 38 million Americans of all ages, and an estimated 529 million people worldwide. It is a significant risk factor for polyneuropathy, eye problems, coronary artery disease (CAD), renal disease, heart disease, stroke, and peripheral artery disease (PAD). Nearly 9 million Americans with diabetes are unaware of their condition and, therefore, do not receive health care to prevent disease progression and complications. With T2DM being a condition that leads to increased demand for health care services due to its long-term, persistent nature and its incremental impact on the body, early diagnosis and timely initiation of effective treatments are essential. Despite the effect of metabolic memory in the development of diabetes-related complications, early intervention helps decelerate disease progression, reduce complications, and ultimately improve survival. Various blood-based biomarkers have been identified, which hold great promise to streamline the mechanisms underlying T2DM and its progression from insulin resistance and prediabetes to diabetes and end-stage diabetes. However, the greatest need is to identify and utilize reliable biomarkers that can help to assess pharmacological treatment response and guide efforts to improve insulin sensitivity, preserve pancreatic beta-cell function, and prevent or delay complications. This review explores the clinical utility of promising biomarkers and assess their potential to support more personalized pharmacological approaches tailored to the individual characteristics of T2DM patients.

## 1. Introduction

The recent International Diabetes Federation (IDF) Atlas (2025) estimates that 1 in 9 adults aged 20–79 is affected by diabetes, with nearly 4 in 10 individuals unaware that they have the condition [1]. T2DM is the 8th leading cause of preventable death and disability worldwide and is a major global risk factor for cardiovascular diseases [2]. It is estimated that T2DM accounts for nearly 90% of the global burden of diabetes and can increase the risk of kidney disease [3], hypertension [4], heart disease [5], vasculopathy [6], and neuropathy [7], often with devastating consequences. According to a report by the World Health Organization (WHO), diabetes and kidney disease are responsible for at least 2 million deaths due to diabetes [8]. Evidence indicates that diabetic patients are nearly twice as likely to have a stroke compared to adults without diabetes [9].

The diagnosis of T2DM is based on current classification criteria, including consistent fasting blood glucose levels greater than 130 mg/dL, glycated hemoglobin A1c (HbA1c) greater than 7% (an average blood glucose level of 154 mg/dL), and lifestyle assessment. Blood glucose levels vary considerably and tend to increase with age. The risk of microvascular and macrovascular complications rises with uncontrolled hyperglycemia [10]. Many neuroendocrine factors are involved in blood glucose regulation, including activation of the sympathetic and parasympathetic nervous systems, pancreatic hormones (insulin and glucagon secretion), hepatic processes (glycogenolysis, gluconeogenesis, glycogenesis), and lipolysis in adipose tissue [11]. Key organs involved in glucose homeostasis include the liver, pancreas, and gastrointestinal tract.

Approximately 90% of patients with hyperosmolar hyperglycemic state (HHS) have a known diagnosis of T2DM [12]. These patients are considered to have decompensated diabetes [13]. Hyperglycemic crises can result from increased gluconeogenesis, elevated glycogenolysis, and decreased glucose uptake in tissues such as the liver, muscles, and fat. These distinct metabolic disruptions can lead to glucosuria, elevated serum osmolality [14], dehydration, impaired renal function [15], and a prothrombotic state [16,17]. HHS is a major cause of morbidity and mortality [18], with contributing factors including severe dehydration, advanced age, and the presence of comorbidities [19].

Many medications can have severe, undesirable effects, and prescription antihyperglycemic drugs are no exception. For instance, sodium–glucose co-transporter-2 (SGLT2) inhibitor-induced glucosuria is associated with osmotic diuresis, lower limb amputation, and diabetic ketoacidosis (due to insulin deficiency), which can lead to diabetic coma or even death. Therefore, to ensure that T2DM patients receiving antihyperglycemic medications achieve maximum therapeutic benefit, effective biomarkers should be used to guide clinical evaluation. This would not only improve patient care but also help identify individuals at greatest risk for drug-induced tissue damage.

In order to identify biomarkers, a search is performed using PubMed, Science Direct, Google, and Google Scholar in tandem to cover the most thorough results. Both Google and Google Scholar are used because of their broad search capabilities for locating various document types: conference papers, patents, government reports and statistics [20,21]. The major keywords used to perform the search are the following: “imaging and circulating biomarkers”, “established biomarkers in diabetes/chronic diseases”, “pharmacodynamic biomarker”, “monitoring biomarkers in diabetes”, “predictive biomarkers in diabetes”, and “safety biomarkers”.

**Figure 1:**
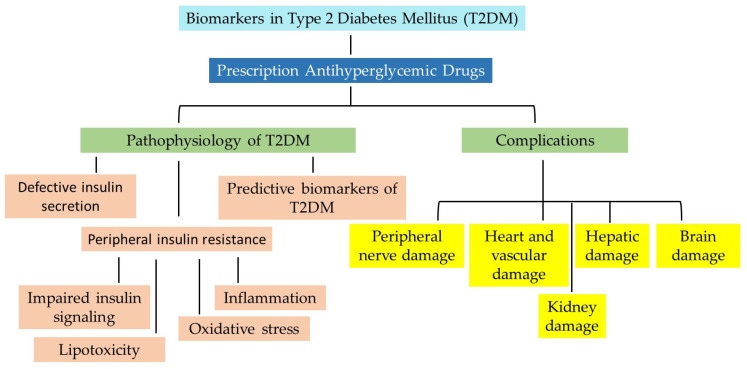
A schematic representation of the main topics discussed in this review.

Therefore, in this review, we examine the applicability of various blood biomarkers studied for their potential to identify and explore the effectiveness, safety, and clinical endpoints of medical treatments in patients with T2DM. A schematic representation of the topics covered in this review is shown in Figure 1.

## 2. Biomarkers Help Monitor Responses to Medications

Potential biomarkers for the risk of diabetes are classified into two categories: genetic and nongenetic [22]. Biomarkers are measurable characteristics found in various biological compartments (blood, fluids, and tissues) in patients, and their diagnostic spectrums differ. While some circulating biomarkers serve as early indicators of improved outcomes and opportunities to optimize dosing in specific populations with diabetes, only a few meet clinical requirements. Abnormal elevation in key biomarkers that signal diabetes may also play a harmful mediating role in type 2 diabetes mellitus (T2DM) and associated cardiovascular risk factors.

Different countries take diverse approaches to diabetes care. However, clinical endpoints meaningful to both clinicians and patients—such as health and well-being—remain essential [23]. Although many biomarkers have been identified over the years, only some consistently predict relevant clinical outcomes in diabetes treatment across different ethnic groups.

Therefore, in this review, we examine the applicability of various blood biomarkers studied for their potential to identify and evaluate the effectiveness, safety, and clinical endpoints of medical treatments in patients with T2DM. A schematic representation of the topics covered in this review is shown in Figure 1.

## 3. Emerging Theories of the Pathophysiology of T2DM

**Figure 2:**
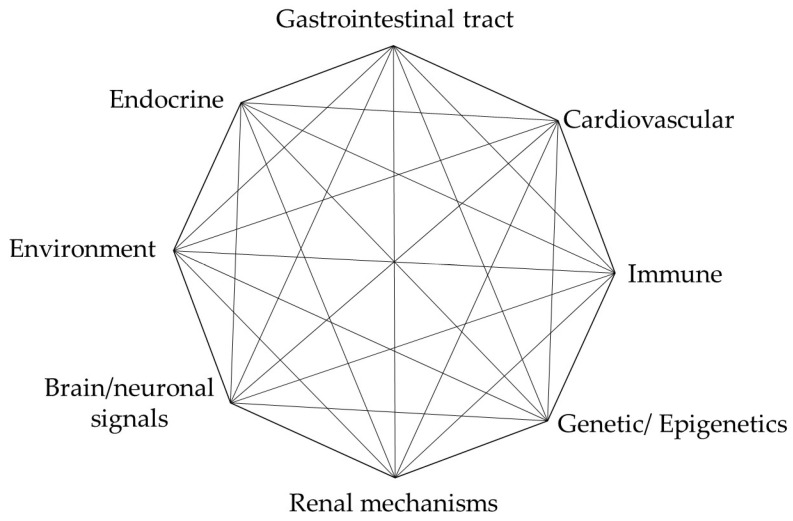
Glucose metabolism and regulation. Network plot illustrating the metabolic fate of ingested carbohydrates and their regulatory sites. The nodes represent systems, tissues, or cellular processes involved in the regulation of carbohydrate metabolism.

T2DM is characterized by the disruption of the blood glucose balance and is emerging as the pathological basis of various diseases, including stroke, cardiac failure, nephropathy, retinopathy, and Alzheimer’s disease. It is a complex and multifactorial disorder involving multiple dysregulated pathways to varying degrees, as illustrated by the nodes in Figure 2.

In the past two decades, new concepts have emerged that have altered our understanding of metabolic interorgan crosstalk and the prevailing roles of pancreatic beta-cells and peripheral insulin resistance in diabetes. As research has developed, the interplay of factors promoting glucose metabolism dysregulation has been demonstrated. While the precise underlying causes of T2DM remain a dilemma, evidence suggests that it is influenced by a myriad of interconnected risk factors such as dysregulated metabolism, impaired vascular function [24], genetic factors [25,26], disruptions or imbalance in epigenetic mechanisms [27,28], dysregulated endocrine function [29], immune dysregulation and inflammatory response [30], disruption of neural signal transmission [31,32,33], and environmental influences including those that disrupt the gut microbiome [34], Figure 2. However, it should be noted that the role of these factors almost certainly varies from one patient with T2DM to the next. For instance, some individuals are obese but do not manifest diabetes or cardiovascular disease.

The heterogeneity of T2DM is mainly due to both defective insulin secretion and peripheral insulin resistance. Upon stimulation, the pancreas is the major organ responsible for releasing digestive enzymes into the gastrointestinal tract and secreting insulin and glucagon into the blood; hence, it plays a critical role in carbohydrate metabolism. Individuals with defective insulin secretion are initially capable of maintaining glucose levels within the normal range via a compensatory mechanism that triggers an increase in insulin secretion. As the disease progresses, beta-cell mass and its ability to maintain insulin production and release change, resulting in imbalanced glucose homeostasis and hyperglycemia.

Insulin resistance is a major cause of T2DM and is characterized by the reduced effectiveness of insulin in triggering cascades of events that enable glucose uptake and metabolism in peripheral tissues such as muscle, fat, and liver, resulting in increased blood glucose levels and compensatory hyperinsulinemia.

The major mechanisms by which insulin resistance develops are four-fold. First, dysfunction in proteins involved in glucose uptake leads to impaired insulin signaling. Second, elevated circulating free fatty acid (FFA) levels result in disturbances in lipid metabolism, which may not only contribute to the development of impaired beta-cell mass and insulin secretion [35,36] but also accumulate in organs such as muscle and liver [37], leading to lipotoxicity and thus promoting T2DM. Third, chronic low-grade inflammation through various inflammatory mechanisms, including lack of physical activity, dyslipidemia, hypertension, abnormal incretin biology, genetic predisposition, and abnormalities in gut microbiota, contributes to insulin resistance. Clinical studies such as CANTOS (Canakinumab anti-inflammatory thrombosis outcomes study) [38], JUPITER (Justification for the Use of Statins in Prevention: an Intervention Trial Evaluating Rosuvastatin) [39], and COLCOT (colchicine cardiovascular outcomes trial) [40] undoubtedly provide significant evidence that chronic inflammation contributes to long-term complications of diabetes, including cardiovascular disease, independent of hyperlipidemia. Fourth, oxidative stress, defined by excess endogenous reactive oxygen species, serves as a key mechanism in insulin resistance by damaging insulin receptors and signaling pathways.

In conclusion, elevated blood glucose levels result from multiple forces acting on cellular networks within and between the gastrointestinal tract, pancreatic beta cells, liver, circulatory system, adipose tissue, kidney, brain, skeletal muscles, and tissue-resident immune cell circuits [41] that regulate systemic metabolism. Dysregulation of these metabolic interorgan crosstalk, due to factors such as inflammation, chronic overnutrition, and aging, contributes to insulin resistance, dyslipidemia, b-cell dysfunction, reduced energy expenditure, liver disease, and metabolic syndrome.

Biomarkers of these interacting pathways are not yet fully characterized in clinical practice and therefore require further validation. Understanding the interactions among the diverse factors that influence the development and progression of diabetes, along with the current knowledge of clinical complications associated with available antidiabetic drugs, can help identify biomarkers that provide valuable insights. These biomarkers have the potential to enable early disease detection, monitor disease progression, and guide more effective, individualized treatment strategies.

In this section, we begin with a brief overview of the history of diabetes management guidelines, highlighting how refinements in our understanding of the nodes shown in Figure 2 have shaped these recommendations. We then summarize the current knowledge of available antidiabetic drug classes that have emerged over the past decade and are likely to influence most nodes presented in the octagonal diagram (Figure 2). While all antidiabetic medications improve HbA1c control and provide cardiovascular and renal benefits, these drugs at normal prescribed doses can still cause harm ranging from mild side effects to severe, life-threatening conditions in a considerable portion of patients, regardless of age or health status. We discuss how the host’s response to antidiabetic drugs induces biological changes in specific pathways of carbohydrate metabolism node axis (Figure 2), involving intricate biochemical processes that determine their potential side effects. Finally, we describe that understanding these interactions is a critical factor in determining the outcome and biomarker choice, while broadening our ability to prevent and mitigate potential side effects.

Here, we briefly discuss the application of this translational paradigm, dating back to the mid-1960s and emphasize the need to identify new predictive biomarkers that reflect disease progression or treatment efficacy.

While the WHO has published guidelines for the diagnosis and classification of diabetes since 1965 [42], it established the first international guidelines for the management of diabetes in 1980. The American Diabetes Association (ADA) published its first Standards of Care in Diabetes clinical practice guidelines a few years later [43]. In defining diabetes, these guidelines have taken two approaches: either basing their definition on a specific threshold for initiating pharmacological treatment or on blood glucose levels above which the risk of complications increases.

The US, Asian, and European guidelines focus on lifestyle changes and have chosen a cut-off level of fasting plasma glucose above which the benefits of treatment, as reported by interventional clinical trials of blood glucose-lowering therapy, are considered to outweigh the harms. However, a thorough risk–benefit evaluation in older adults with T2DM should be performed to assess the potential advantages and disadvantages of treatments, due to the U-shaped relationship between HbA1c and the risk of mortality [44]. Using this approach, the cut-off point that defines diabetes is glycated hemoglobin (HbA1c) <7%, using standard clinical methods of measurement [45].

**Figure 3:**
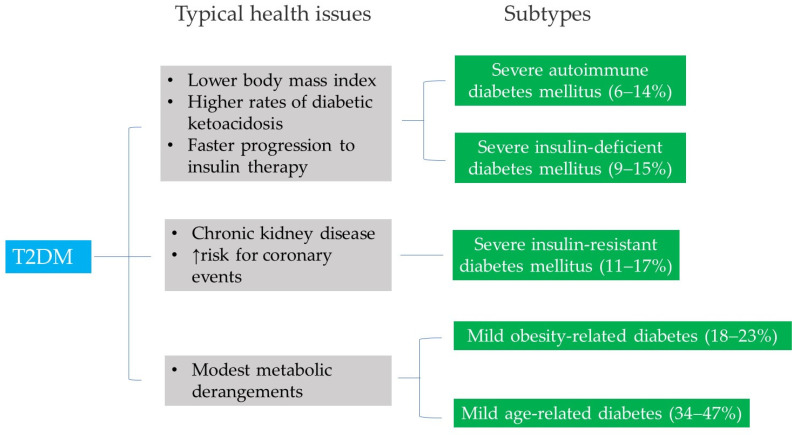
Simplified schematic representation of five different subtypes of diabetes and their association with a range of health issues in patients with T2DM.

Traditionally, T2DM is characterized by a gradual decline in insulin secretion from the pancreas, against a background of insulin resistance. Recently, modifications have been made regarding the diagnosis of impaired fasting glucose [46]. This study emphasizes the range of severity in T2DM by stratifying patients into five subgroups with differing disease progression and risks of diabetes complications (Figure 3). The classification includes five clinical subgroups:

- Subtype 1—Severe autoimmune diabetes mellitus.
- Subtype 2—Severe insulin-deficient diabetes mellitus.
- Subtype 3—Severe insulin-resistant diabetes mellitus.
- Subtype 4—Mild obesity-related diabetes.
- Subtype 5—Mild age-related diabetes.

Severe autoimmune diabetes mellitus (Subtype 1) and severe insulin-deficient diabetes mellitus (Subtype 2) are the least common subtypes, representing 6–14% and 9–15% of the study cohorts, respectively [46]. While individuals in Subtype 1 are positive for autoimmune response, those in Subtype 2 exhibit inadequate insulin production. Both subtypes share characteristics similar to type 1 diabetes mellitus, including lower body mass index, higher rates of diabetic ketoacidosis, and faster progression to insulin therapy.

Individuals with severe insulin-resistant diabetes mellitus (Subtype 3), characterized by insulin resistance, represented 11–17% of the study cohorts. They had a higher risk of progression to chronic kidney disease and were prone to increased risk for coronary events.

Mild obesity-related diabetes (Subtype 4) and mild age-related diabetes (Subtype 5) are the most common subtypes, representing 18–23% and 34–47% of the study cohorts, respectively. Individuals in these two subtypes tend to experience metabolic derangement [47]. This suggests that individual metabolites and a metabolomic network are significantly associated with T2DM in these two cohorts.

The findings of this study change the definition of T2DM by emphasizing its heterogeneity. This suggests that to effectively manage T2DM subgroups, a new approach is essential—one that emphasizes a comprehensive understanding of biomarkers and targeted interventions to optimize care.

A link between glycemic control and diabetes complications was initially recognized in the late 1980s [48,49,50]. The benefit of lowering blood glucose to prevent diabetes-related complications and reduce cardiovascular events and mortality is unequivocal in patients with substantial elevations in blood glucose levels. Over two decades ago, the Diabetes Control and Complications Trial (DCCT) [51] on patients with type 1 diabetes and the United Kingdom Prospective Diabetes Study (UKPDS) [52] on patients with T2DM reported that maintaining optimal HbA1c levels, an indicator of mean glycemia, helps prevent disease progression and is crucial for reducing the risk of macrovascular and microvascular complications.

The first randomized controlled interventional study of anti-diabetes therapy in patients with elevated blood glucose levels is shown in the DCCT report. Subsequent studies, which have mostly used measures of clinic blood glucose levels, have shown the benefit of glucose-lowering therapy when HbA1c is above 7% in all patients up to age 80 years [53,54,55], with a caveat: severe hypoglycemia is a frequent event in the elderly population, and increased mortality risk becomes apparent among those with HbA1c values of 6.4% [56].

The Action to Control Cardiovascular Risk in Diabetes (ACCORD) trial elegantly demonstrated the benefits and risks of intensive glucose control, intensive blood pressure control, and lipid management in high-risk patients with T2DM [57]. The ACCORD trial showed the highest burden of excess deaths at both extremes of HbA1c compared to the average HbA1c level for adults with diabetes. HbA1c can be highly variable within an individual and is not well characterized from a single or a limited number of measurements. Moreover, HbA1c levels vary disproportionately in certain groups of patients with T1DM [58,59] and T2DM [59,60,61].

Thus, recent guidelines have redefined hyperglycemia based on the degree of HbA1c associated with increased cardiovascular risk. They recommend individualization of HbA1c targets to reduce the risk of cardiovascular events [62,63]. While HbA1c is considered efficient in assessing the prevalence of pre-diabetes and undiagnosed diabetes [64], and a reliable marker for assessing long-term blood glucose control, it is influenced by numerous factors, including population differences, hemoglobin variants, anemia, and other medical conditions.

Studies also suggest that T2DM is a broad term encompassing a complex range of conditions that affect blood glucose regulation [5] (Figure 3). Since individual treatment decisions are more complex than a definition, new biomarkers and targeted interventions are essential for advancing treatment strategies in patients with T2DM and in critically ill diabetic patients.

## 4. Potential Biomarkers of Adverse Drug Reaction for Oral Antidiabetic Medications

HbA1c levels are crucial to diabetes management, as persistent hyperglycemia not only promotes advanced glycation end-product (AGE) formation, but also induces oxidative stress and chronic low-grade inflammation, the two major factors involved in vascular complications [17]. Longitudinal HbA1c trends in patients with diabetes exhibit unstable patterns, including increasing, decreasing, and non-linear behaviors [65,66]. These variations are influenced by multiple factors such as age, gender, ethnicity, diabetes duration, disease management frequency, cardiovascular risk factors, and family environment [26]. For instance, patients with T2DM who maintain a mean HbA1c between 6 and 8% can significantly lower the risk of major cardiovascular events [67]. However, tilting HbA1c levels in high-risk patients to values below 7% or values above 8% can lead to poorer outcomes for the patients, indicating the presence of intricate systems, each governed by a delicate balance of hormones.

Despite the availability of oral antihyperglycemic agents, evidence-based guidelines, and HbA1c monitoring, 50% to 70% of people with T2DM globally fail to achieve recommended HbA1c targets [68]. Thus, identifying and applying novel biomarkers is essential to better assess disease risk, predict therapeutic response, and improve clinical outcomes in diabetic patients.

While antihyperglycemic medications generally offer similar therapeutic efficacy and safety, each class may present unique risks due to specific effects on intracellular signaling or insufficient research on their mechanisms. Identifying these drug-specific risks not only supports dose-to-function analysis but may also reveal previously uncharacterized metabolic pathways, offering deeper insights into drug–cell interactions. Taken together, these risk profiles could serve as early biomarkers to predict physiological responses to new or combination therapies, particularly in sensitive populations such as children, pregnant women, and high-risk patients. This section addresses a practical review of current evidence on biomarkers associated with various antidiabetic drug classes and the biological pathways they affect, with emphasis on comorbidities and the pathophysiological understanding of these processes. It also summarizes data on the risks of antidiabetic medications, which can range from mild to severe and occasionally life-threatening. Furthermore, it highlights the critical role of biomarkers as indispensable tools in drug development, treatment response prediction, and the overall improvement of patient outcomes.

### 4.1. Mechanisms of Action, Benefits, and Risks of Oral Antihyperglycemics

#### 4.1.1. Metformin

The first-line treatment option, metformin, is safe and fairly well-tolerated, has excellent long-term effectiveness. It reduces glucose production in the liver (gluconeogenesis) and improves insulin sensitivity in peripheral tissues. Metformin produces both AMP-activated kinase (AMPK) dependent and independent effects [69]. It offers potential benefits against heart failure, neuropathy, and retinopathy, and remains safe [69]. However, meta-analyses cast a shadow on the effectiveness of metformin in reducing the risk of adverse cardiovascular outcomes [70].

**Figure 4:**
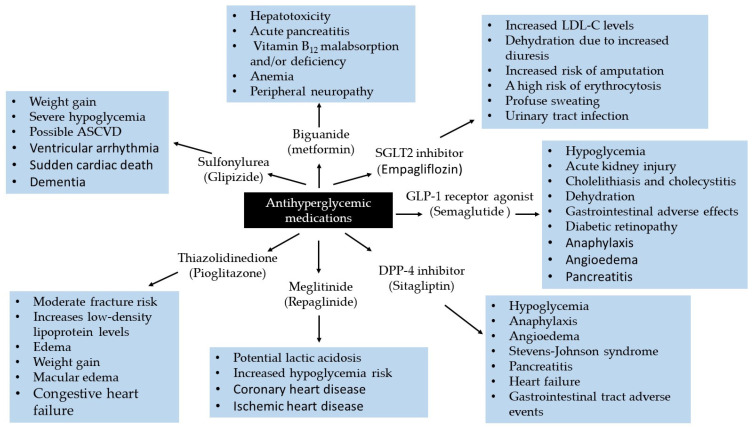
Complications associated with the use of antihyperglycemic drugs.

As demonstrated in Figure 4, while metformin plays a pivotal role in managing hyperglycemia, it is associated with several side effects. Notably, it can induce lactic acidosis in patients with eGFR less than 30 mL/min/1.73 m^2^ [71,72]. Metformin use has also been implicated with serious conditions such as hypotension [73]. However, recent evidence indicates that oral metformin attenuates the hypotensive response to meals [74]. Interestingly, recent evidence suggests that metformin-induced peripheral neuropathy is dose-dependent [75]. In some cases, metformin has been reported to affect vitamin B_12_, the blood coagulation system, hepatotoxicity, or acute pancreatitis [76]. The U.S. Food and Drug Administration (FDA) has retained warnings regarding acute or unstable congestive heart failure on metformin therapies used for T2DM. Metformin remains contraindicated in patients with severe renal impairment due to associated cardiovascular concerns [77].

##### Mechanism by Which Metformin May Cause Side Effects

Metformin-induced vitamin B_12_ malabsorption is mostly seen in elderly individuals and in select pediatric cases of non-diet-induced B_12_ deficiency. Evidence shows that microbiota, nutrients, and metformin can interact through crosstalk among the gut–brain–kidney axis to modulate the homeostasis of bioactive molecules, systemic inflammation, and energy metabolism [78]. While numerous hypotheses have been proposed regarding the mechanisms underlying vitamin B_12_ deficiency in patients treated with metformin, the most widely accepted explanation is that metformin-induced B_12_ malabsorption results from its interaction with calcium in the ileum, the primary site of vitamin B_12_ absorption [79,80]. Studies have demonstrated that co-administration of metformin and calcium can restore vitamin B_12_ bioavailability compared with metformin alone [81].

Because the neurological manifestations of untreated vitamin B_12_ deficiency can lead to nerve and brain damage as well as anemia, patients receiving metformin should be regularly evaluated for vitamin B_12_ deficiency. Metformin has a well-established glucose-lowering effect and is capable of reducing microvascular complications; however, it is crucial to understand its potential adverse effects, particularly its impact on idiosyncratic hepatotoxicity, vitamin B_12_ deficiency, anemia, and lactic acidosis. These side effects remain major concerns for both patients and clinicians. While the automated red blood cell counts and hematocrit values are imported, cytokine testing should also be considered in diabetic patients treated with metformin.

Furthermore, evidence suggests that the microbiota are altered during aging and in age-related diseases [82], emphasizing the importance of the complex interplay between metformin treatment, diet, and gut microbiota. These findings highlight the potential benefits of optimizing metformin use to mitigate its possible side effects.

#### 4.1.2. Insulin-Releasing Medications

##### Sulfonylureas

Sulfonylureas have the capability to lower blood glucose levels [83] and are associated with the reduced risk of diabetic retinopathy [84], indicating a beneficial microvascular effect. Their effects are primarily due to stimulation of insulin secretion and suppression of hepatic glucose output [85], leading to a reduction in the glycation of hemoglobin A1c. Effective elevation in insulin sensitivity has also been reported at peripheral target sites [86]. While hypoglycemia is associated with first- and second-generation sulfonylureas due to their strong binding to blood carrier proteins, glimepiride (the third-generation sulfonylureas) poses a lower risk of hypoglycemia because of its reduced protein binding. WHO and the International Diabetes Federation (IDF) recommend sulfonylureas as an option after metformin or in combination with drugs such as SGLT2 inhibitors or dipeptidyl peptidase-4 (DPP-4) inhibitors [87]. However, they fail to stimulate insulin release in individuals with certain mutations in the KCNJ11 gene [26].

While generally well-tolerated, sulfonylureas may elicit adverse effects, including microvascular complications [88], cardiovascular events and mortality [89], myocardial infarction [90], ventricular arrhythmias and sudden cardiac death [91], severe hypoglycemia, atherosclerosis, cardiovascular disease (ASCVD), and dementia (Figure 4).

##### Meglitinide

Postprandial glucose has been recognized as a greater risk factor for cardiovascular disease than fasting plasma glucose for over two decades [92,93]. Meglitinides are one of the commonly prescribed classes of anti-hyperglycemic agents that trigger pancreatic beta cells to release insulin [94] in order to target postprandial glucose excursions [93]. The release of insulin occurs via the inhibition of adenosine triphosphate (ATP)-dependent potassium channels, leading to depolarization of beta cells [95,96].

Repaglinide, a meglitinide, decreases HbA1c by 0.7% in patients with T2DM [97]. Repaglinide improves homocysteine, plasma activator inhibitor, and lipoprotein (a) [98]. Improved glucose metabolism is recognized as a major driving force in reducing these metabolic parameters. Repaglinide is inactivated in the liver and primarily excreted via the bile, and as such, its excretion is not affected by renal disorders. This class of drug is a safe antidiabetic option for the elderly [99] and renal impaired patients [94]. Patients who cannot tolerate metformin can switch to repaglinide.

Despite being an effective treatment option, there are risks associated with repaglinide therapy (Figure 4). It has been linked to an increased cardiovascular event risk [100], including coronary heart disease, ischemic heart disease [101]. Unlike metformin, repaglinide may increase the risk of major adverse cardiovascular events (MACE), as reported in a Danish nationwide registry-based observational analysis [98].

##### Cardiovascular Risks and Mechanistic Insights into Insulin Secretagogues

The sulfonylureas and glinides are characterized as insulin secretagogues. A meta-regression analysis indicates that sulfonylurea monotherapy is associated with a higher risk of cardiovascular events and mortality [89,91] compared with glinides [98]. Sulfonylureas are among the most widely prescribed oral antidiabetic drugs. It should be noted that their adverse effects are not class effects but are generally observed at the level of individual sulfonylurea compounds.

The DIGAMI (Diabetes Mellitus, Insulin–Glucose Infusion in Acute Myocardial Infarction) study provided evidence that intensive insulin treatment does not result in better cardiovascular outcomes compared with sulfonylurea therapy [102]. Cardiac and skeletal muscle [103], endothelial cells, and the brain [104] express the sulfonylurea receptor 2 (SUR2) subtype, which forms a complex with glucose transporters. Deletion of cardiomyocyte SUR2 results in enhanced glucose uptake and protects the heart from myocardial ischemia–reperfusion injury [105]. However, a single Kir6.1 variant has been associated with J-wave syndrome, which is characterized by electrocardiographic abnormalities [106]. Moreover, mutations in the SUR2 gene are linked to Cantu syndrome. It has been discovered that sulfonylureas act as partial antagonists that inhibit the SUR2 subtype of cardiomyocyte KATP channels [107].

The clinical relevance of the DIGAMI study and related preclinical observations has led to the hypothesis that inhibition of SUR2 could potentially impair coronary vasodilation [108] and increase the risk of sudden cardiac arrest through effects on both SUR1 and SUR2 [109]. Many clinical and pharmacological aspects of SUR2 are discussed in greater depth in these reports [109,110].

Molecular biomarkers (adipokines, sortilin, klotho and FGF23, brain natriuretic peptides, troponins) [111] and vascular imaging of atherosclerosis [112], which can evaluate biological processes at the molecular and tissue levels within the cardiovascular system, are indispensable tools for monitoring treatment effectiveness and predicting patient outcomes.

#### 4.1.3. Thiazolidinedione

Among the therapeutic options for the treatment of hyperglycemia, thiazolidinediones (TZDs) represent a significant breakthrough in the history of diabetes management, preceding the discovery of DPP-4 inhibitors, SGLT2 inhibitors, and glucagon-like peptide-1 receptor (GLP-1R) agonists. Pioglitazone, a TZD, directly improves insulin sensitivity [113], effectively lowers HbA1c [114], provides durable glycemic effects, and carries a low risk of hypoglycemia [115]. Pioglitazone has demonstrated benefit in patients with ASCVD and those at risk for stroke [116,117,118]. Pioglitazone can effectively serve as a valuable second-line agent with unique cardiovascular advantages [116].

##### Understanding the Off-Target Effects of Thiazolidinedione

Like sulfonylureas, the risks associated with TZDs are drug-specific and not class effects. The side effects of TZDs can arise for various reasons, including incorrect dosage, drug interactions, duration of use, combination therapy with insulin, and individual differences in drug metabolism. While some adverse effects are preventable, increased fracture risk, fluid retention, and potential hepatotoxicity remain significant concerns (Figure 4).

There is an association between the duration of T2DM and fracture risk. Microvascular complications, along with poorer glycemic control, are considered two major factors contributing to elevated fracture risk [119,120]. Preclinical studies show that both rosiglitazone and pioglitazone cause bone loss by inhibiting osteoblast activity and bone formation [121]. Osteocalcin, bone-specific alkaline phosphate, carboxy-terminal propeptide of type 1 collagen, and aminoterminal propeptide of type 1 collagen are commonly used as markers of bone formation [122]. These biochemical markers of bone turnover may help assess and potentially prevent fracture risk in diabetic patients treated with pioglitazone.

Evidence also indicates that fluid retention is associated with an increased risk of both ASCVD and venous thromboembolism. Fluid retention and edema are attributed to the salt-retaining effects of PPARγ activation on nephron ion transporters [123]. Beltowski et al. [123] provide additional background on thiazolidinedione-induced fluid retention. The heart, kidney, and lung are all involved in volume regulation. Since heart biomarkers (e.g., brain natriuretic peptide) and kidney biomarkers (e.g., kidney injury molecule-1) are affected by renal insufficiency, organ failure markers should be considered when TZDs are administered to patients.

#### 4.1.4. Dipeptidyl Peptidase-4 (DPP-4) Inhibitors

DPP-4, an endogenous aminopeptidase enzyme, metabolizes incretin hormones, resulting in decreased insulin secretion. DPP-4 exists in two isoforms [124]: one is free in the plasma and the other is anchored in the membrane of numerous cell types, including endothelial cells, T cells, and kidney tubular cells [125]. While circulating DPP-4 is responsible for the metabolism of circulating GLP-1, membrane-bound DPP-4 appears to be involved in the cleavage of several other substrates, including pro-brain natriuretic peptide, neuropeptide Y, and stromal cell-derived factor-1α [126].

The two incretin hormones, GLP-1 and glucose-dependent insulinotropic polypeptide (GIP), play key roles in the pathophysiology of both obesity and T2DM. Elevated concentrations of incretin hormones lead to increased insulin secretion, decreased glucagon release, and reduced hepatic glucose production [127]. GLP-1 and GIP exert insulinotropic and glucagonostatic effects [128] via the GLP-1 receptor—and GIP receptor-mediated stimulation, thereby maintaining optimal plasma glucose levels. While the biological effectiveness of GIP in inducing insulin secretion by pancreatic β-cells is significantly reduced in T2DM patients, the insulinotropic and glucagonostatic effects of GLP-1 remain largely preserved [127].

Evidence confirms a prominent role of DPP-4 in the pathophysiology of T2DM [129]. DPP-4 inhibitors are capable of modulating several interrelated biochemical pathways to decrease glucagon and postprandial glucose levels, promote satiety, and improve glycemic control [130]. However, the role of DPP-4 inhibitors in delaying gastric emptying remains debated, with studies reporting varying outcomes [130,131]. Clinical studies further provide evidence that DPP-4 inhibitors may exert both anti-inflammatory and immunomodulatory effects [132] and exhibit protective actions against the progression of renal disease [133,134].

Notably, recent data mining from the public version of the Food and Drug Administration (FDA) Adverse Event Reporting System (FAERS) indicates that sitagliptin, saxagliptin, linagliptin, and vildagliptin (a DPP-4 inhibitor) are disproportionally associated with gastrointestinal nonspecific inflammation and dysfunction, pancreas, and musculoskeletal disorders [135] and upper respiratory infection [136]. A meta-analysis of cardiovascular outcome trials provides supporting evidence that DPP-4 inhibitors may increase the risk of atrial flutter [137]. Saxagliptin, in particular, has been repeatedly associated with an increased rate of hospitalization for heart failure [138,139], although this association remains debated due to the insufficient number of large-scale trials [140] or selection of diabetic patients with differing CV risk categories, which does not align with the 2019ESC-EASD guidelines (very high risk, high risk, moderate risk) [141].

##### DPP-4 Inhibitor: Insights into Preventing the Adverse Effects

Studies indicate that inhibitors of DPP-4 may be involved in the development of arthritis [142,143,144]. DPP-4 inhibitors have been associated with arthritis and arthralgia regardless of the specific agent used, suggesting a possible class effect [143,145]. Evidence also suggests that HLA-DRB3, an antigenic peptide, could serve as a potential genetic marker for the early detection of rheumatoid arthritis (RA) and for evaluating treatment efficacy [146]. Although no significant increase in the risk of musculoskeletal conditions has been observed among DPP-4 inhibitor users [147], their use has been linked to acute pancreatitis [148]. Therefore, measurement of pancreatic enzyme levels and abdominal ultrasonography are recommended to exclude pancreatic injury.

#### 4.1.5. Glucagon-like Peptide-1 (GLP-1) Agonist

GLP-1-induced activation of the GLP-1 receptor (GLP-1R) triggers a complex intracellular signaling cascade that finally activates the protein kinase A (PKA) pathway through the production of cyclic adenosine monophosphate (cAMP) [149]. GLP-1 agonists are associated with weight loss and a low risk of hypoglycemia.

GLP-1 agonists are recognized as neuroprotective and cardioprotective agents by reducing inflammation, stimulating nerve growth and affecting lipid metabolism [150]. GLP-1 is capable of lowering glucose through multiple mechanisms, including insulin release stimulation and suppression of glucagon secretion [151]. Thus, GLP-1 agonists offer both cardioprotective and neuroprotective benefits. Recently, a systematic review and meta-analysis of randomized clinical trials reported that semaglutide may elevate the risk of diabetic retinopathy [152], suggesting that this medication might worsen microvascular complications in some patients.

##### Insights into Potential Mechanisms of GLP-1R Agonist Side Effects

GLP-1R agonists represent a promising therapeutic option for metabolic diseases. They have demonstrated benefits in reducing the risk of several conditions, including cardiovascular disease, nonalcoholic steatohepatitis (NASH), Alzheimer’s disease (AD), and chronic kidney disease. However, studies suggest that GLP-1R agonists may be associated with a higher risk of neovascular age-related macular degeneration [153] and diabetic retinopathy [154]. Many clinical and pharmacological aspects of GLP-1R agonists have been discussed in greater depth in recent reviews [155]. Further studies are needed to clarify the risk of visual deterioration associated with GLP-1R agonists and to determine whether this risk extends across the entire GLP-1R agonist class.

#### 4.1.6. Sodium–Glucose Co-Transporter-2 Inhibitors

SGLT2 inhibitors reduce blood glucose by increasing urinary glucose excretion [151] and improve cardiovascular and renal outcomes, especially in patients with high cardiovascular risk, as demonstrated in EMPA-REG OUTCOME and other trials [156,157]. SGLT2 inhibitors, also known as gliflozins [158], reduce HbA1c by 0.5–1.0%, enhance insulin sensitivity, and exhibit pleiotropic benefits, including diverse cardiovascular- and kidney-protective effects [158,159,160]. SGLT2 inhibitors have been shown to lower hyperuricemia, epicardial fat mass, as well as oxidative stress [156]. They also promote autophagy and lysosomal degradation, and contribute to the elevation in erythropoietin levels via SIRT1-dependent mechanisms [161], increase circulating pro-vascular progenitor cells [162], and improve vascular function.

In summary, although numerous classes of glucose-lowering drugs are widely available, most patients do not achieve optimal glucose control. Evidence indicates that glucose management should focus not only on reaching and maintaining optimal HbA1c levels but also on reducing and preventing the long-term complications of diabetes [163,164]. Recent guidelines highlight the association between LDL-C and ASCVD, which encompasses a range of clinical manifestations such as acute coronary syndrome, myocardial infarction, stable or unstable angina, coronary or other arterial revascularization, stroke, transient ischemic attack, and PAD [165,166,167,168].

There is a relationship between the atherogenic index of plasma (AIP) and chronic microvascular complications in individuals with T2DM. AIP serves as an indicator of disturbed plasma lipoprotein metabolism and reflects the presence of inflammation [169,170]. Indeed, while all new antidiabetic medicines are beneficial for blood sugar management, agents such as metformin, sulfonylureas, DPP-4 inhibitors, GLP-1R agonists, and SGLT2 inhibitors offer varying degrees of cardiovascular and renal protection, none have demonstrated efficacy against other complications such as retinopathy, peripheral neuropathy, diabetes-associated liver disease (e.g., nonalcoholic fatty liver disease, NAFLD), or diabetes-associated inflammatory disorders. Moreover, none of the current therapeutic approaches appear effective in delaying the progression from prediabetes to diabetes or to severe diabetes.

This underscores the complex interplay between the elusive nature of diabetes, T2DM medications, and blood glucose regulation, emphasizing the need for a deeper understanding of the mechanisms underlying hyperglycemia, insulin resistance, and β-cell dysfunction, as well as the identification of reliable biomarkers for assessing cardiovascular risk and predicting serious side effects.

Antihyperglycemic drugs can affect multiple organ systems, with side effects ranging from mild to life–threatening, highlighting the need for ongoing post-marketing surveillance. Table 1 summarizes serious adverse events for each antidiabetic medication and presents potential predictive biomarkers that may help anticipate complications, guide therapy, and identify patients at risk for treatment-related pathological conditions.

In summary, although extensive research has elucidated the mechanisms underlying the clinical hazards of antidiabetic drugs, in this section, we have aimed to present recent understanding and accepted knowledge about the functions and benefits of these medications, with special emphasis on collating outcomes related to their multiorgan toxicities. We have also summarized and proposed potential predictive biomarkers based on recent findings aligned with emerging pathways in the pathogenesis of diabetes-related complications. Our focus has been on the detrimental impacts of antidiabetic drugs on eight major organs (Table 1) whose coordinated functioning is essential for glucose metabolism, regulation, and overall health.

**Figure 5:**
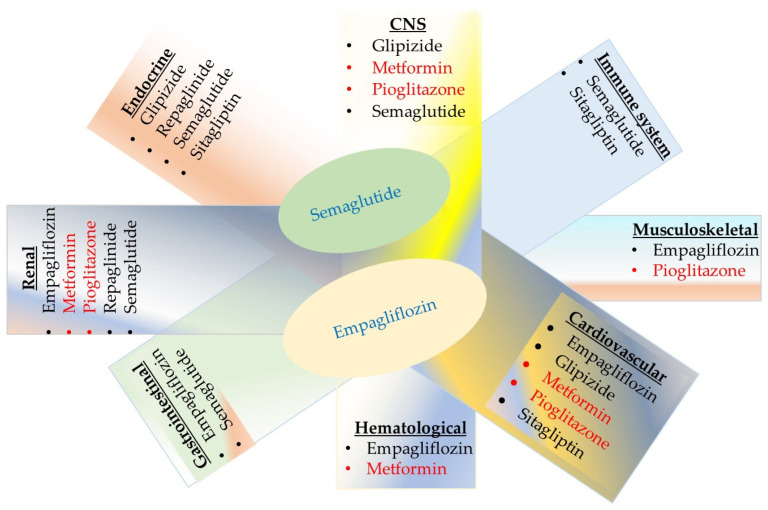
A schematic representation summarizing the serious side effects of antidiabetic drugs on the human body. Eight nodes (underlined in the figure) participate in the regulation of carbohydrate metabolism, and two commonly used traditional antidiabetic drugs shown in red negatively affect four of these nodes.

A schematic representation summarizing the serious side effects of antidiabetic drugs on the human body is presented in Figure 5. While all have a generally favorable benefit–to–risk profile, there are serious risks associated with both traditional antidiabetic drugs (metformin, glipizide, repaglinide, and pioglitazone) and newer agents (semaglutide, sitagliptin, and empagliflozin). Medications exhibiting serious toxic effects on four or five organ systems are highlighted in red or blue, respectively. In addition to gastrointestinal and renal complications, empagliflozin carries risks of musculoskeletal, hematological, and cardiovascular side effects. Semaglutide is linked to serious complications involving the central nervous, endocrine, immune, gastrointestinal, and renal systems. Since diabetes has a genetic component and the pharmacogenomics of diabetic patients remains largely unknown, individualizing drug therapy based on a patient’s genetic and genomic profile to prevent adverse reactions is currently not feasible. Therefore, clinicians should monitor the function of cardiovascular, renal, immune, endocrine, musculoskeletal, hematological, and gastrointestinal systems, along with patient-reported side effects.

## 5. Emerging Biomarkers in T2DM

While growing evidence supports the utility of biomarkers in improving diabetes diagnosis, the prognostic value of only a few biomarkers appears to influence therapy, and current guidelines do not recommend their routine measurement for risk stratification or to assess the comparative effectiveness of dosing and combination therapies in people with T2DM. A recent systematic review indicates that changes in hematological protein, cytokine, and lipid profiles are evident in T2DM patients [250].

### Markers of Chronic or Low-Grade Inflammation

In T2DM, obesity-induced macrophage infiltration causes low-grade inflammation, leading to the release of proinflammatory mediators [251]. Interleukin-6 (IL-6) is produced by activated leukocytes, endothelial cells, and adipocytes [252], whereas C-reactive protein (CRP), an acute-phase plasma protein, is synthesized by the liver and released into the bloodstream in response to IL-6 exposure [253]. CRP exists in two isoforms with opposing actions: native pentameric (pCRP), an anti-inflammatory form, and monomeric (mCRP), a proinflammatory form [254,255]. mCRP has been found to cause platelet activation [256,257], leukocyte recruitment [256], and endothelial dysfunction [258], all of which are implicated in the pathogenesis of various diseases, including diabetes. IL-6 and monomeric CRP, two of the most commonly assayed inflammatory biomarkers, are used to refine diabetes risk prediction. IL-6 primarily reflects the severity of inflammation, the link between inflammation and diabetes, and the prevalence of associated diabetic complications.

However, CRP levels may be elevated due to various conditions, including both acute and chronic responses to infection, noninfectious triggers, or factors such as moderate-to-vigorous physical activity and increased sedentary time [254]. Neither IL-6 nor CRP levels alone accurately reflect the diabetic patient’s response in terms of vascular health (e.g., atherosclerosis, insulin resistance), changes in hematological parameters, dyslipidemia, beta (β) cell responsiveness, cardiovascular disease (CVD), or variability in drug response. The MASCADI (Arachidonic Acid Metabolism in Carotid Stenosis Plaque in Diabetic Patients) study demonstrated a significant elevation in 2-arachidonoyl-lysophatidylcholine (2-AA-LPC) in plaques from diabetic patients, highlighting its potential role in diabetic atherosclerosis as suggested by the authors [259]. Evidence indicates that high sensitivity C-reactive protein (Hs-CRP) may be associated with the metabolic variables and predictors of cardiovascular risk in T2DM with and without nephropathy [260,261]

Notably, the impact of the IL-6 pathway on diabetes risk in the general population appears to be small [262]. Similarly, CRP is not considered an independent risk factor for T2DM [263]. However, CRP has shown a statistically significant association with a favorable effect of metformin in T2DM patients [264].

Destruction of healthy tissue due to disease activates the body’s immune system. This activation, in turn, causes inflammation and irritation in blood vessels and can be seen across a variety of tissues. Chronic inflammation, a low-grade and persistent form of inflammation, is involved in the development and progression of autoimmune diseases and metabolic disorders such as atherosclerosis and obesity, two major global health problems [261].

An emerging hypothesis suggests that part of the inflammatory response observed in T2DM may be attributable to an autoimmune phenomenon, as reactive autoantibodies against islet antigens have been detected in a subset of patients [2]. While T2DM has generally been considered a metabolic disease and metabolic determinants, recent studies have mainly focused on cells of the innate immune system [265,266], and particularly the direct involvement of acquired immunity. Macrophage, monocytes, and lymphocytes are major cells of the innate immune system, and play a significant role in the regulation of inflammation [267].

Obesity is considered a significant risk factor in the development and progression of T2DM [268]. In turn, both T2DM and obesity promote atherosclerotic changes through metabolic and inflammatory mechanisms. The metabolic complications of obesity, such as impaired glucose tolerance, insulin resistance, and lipotoxicity-induced β-cell dysfunction, are collectively referred to as metabolic syndrome, which is associated with a proinflammatory state [269]. Notably, there is a close interplay among metabolism, diet, the immune system, adipose tissue, and contribute to inflammation and disease [270,271].

Insulin resistance, a key metabolic complication, triggers mitochondrial dysfunction, leading to tau protein aggregation and inhibition of lipolysis through a cascade of cellular events. Persistent inflammation is an important contributor to a wide range of diseases [272,273], including T2DM, through mechanisms involving insulin resistance and islet β-cell failure [265]. T2DM-associated vascular disease can result in vascular dementia [274] and is recognized as a risk factor for neurological conditions such as AD. Other factors, such as age [275], genetics [276], lifestyle [277], and cardiovascular diseases like hypertension [278] may also increase the risk of vascular dementia. However, no genetic evidence for a causal relationship between T2DM and AD has been found [279,280]. One possible explanation is that neither T2DM nor AD is a monogenic disorder.

Patients with T2DM often exhibit dysregulated cytokine production and chronic inflammation, reflecting an impaired immune response unable to prevent persistent inflammatory alterations. Several recent reviews have discussed the roles of inflammation and immune dysregulation in influencing disease susceptibility and overall health [281,282].

## 6. Vascular Complications Associated with T2DM

Hyperglycemia-induced vascular injury in type 2 diabetes mellitus (T2DM) is classified into two categories: microvascular and macrovascular complications (Figure 1). Individuals living with diabetic panvascular disease are markedly more susceptible to a broad spectrum of microvascular complications affecting organs such as the heart, brain, eyes, and kidneys. Moreover, they face an increasing risk of developing macrovascular complications. The macrovascular triad in diabetes, encompassing coronary artery disease, cerebrovascular disease, and PAD, contributes substantially to morbidity and mortality. These widespread vascular impairments lead to poor circulation, a strong predictor of cardiovascular mortality, emphasizing the urgent need for early detection and precise prognostic tools. Biomarkers reflecting the presence and severity of vascular complications in diabetes have shown great promise in improving prognostic accuracy. This section will examine both microvascular and macrovascular complications of uncontrolled T2DM, with a particular focus on emerging biomarkers for the non-invasive diagnosis and treatment of diabetes.

### 6.1. Microvascular Complications

#### 6.1.1. Retinopathy

Diabetic retinopathy, a common detrimental microvascular complication of diabetes, involves damage to the blood vessels within the retina [283]. It is a progressive condition that can worsen over time and lead to significant visual impairment or complete vision loss if not adequately treated [284]. Persistent long-term hyperglycemia contributes to the development of diabetic retinopathy by inducing morphological, structural, and functional changes in the small retinal blood vessels, a condition known as microangiopathy [285,286]. If left untreated, this damage can lead to various clinical manifestations.

Hyperglycemia is the main driver in the development of non-proliferative diabetic retinopathy (NPDR), which is characterized by microaneurysms, retinal hemorrhages, and retinal exudates [287]. It can progress to proliferative diabetic retinopathy (PDR), the most advanced stage of diabetic eye disease in T2DM, marked by the proliferation of abnormal new blood vessels on the surface of the retina [288]. This advanced stage poses a serious risk of significant visual impairment.

While genetic predisposition may influence an individual’s vulnerability to retinopathy [289], several risk factors, including the duration of diabetes, poor glycemic control, hypertension, and dyslipidemia, are also associated with the development and progression of diabetic retinopathy [290].

##### Potential Biomarker Options for Retinopathy

Proangiogenic agents

Endothelial peroxisome proliferator-activated receptor γ coactivator (PGC1α)-mediated activation of estrogen-related receptor alpha (ERRα) under hypoxic conditions leads to the stimulation of angiogenic factor expression, which in turn triggers angiogenesis [291]. This signaling pathway also plays a significant role in regulating numerous genes involved in fatty acid metabolism and oxidative phosphorylation in the adult myocardium [292], as well as mitochondrial regulation [293]. Notably, this pathway has been identified as a promising therapeutic target in the treatment of diabetes [294], underscoring the pleiotropic nature of PGC1α. Interestingly, analysis of vitreous fluid samples from patients with proliferative diabetic retinopathy (PDR), non-diabetic individuals, and epiretinal fibrovascular membranes from PDR patients suggests that the PGC1α/ERRα pathway is suppressed in individuals with PDR [295].

Erythropoietin, a potent angiogenic factor induced by ischemia, appears at higher concentrations in the eyes of patients with diabetic macular edema compared to those with age-related macular degeneration (AMD) or non-diabetic individuals [296].

Proinflammatory agents

Patients with diabetic retinopathy exhibit elevated serum levels of irisin and intercellular adhesion molecule-1 (ICAM-1) in the early stages of the disease, with levels decreasing in later stages [297]. These molecules may serve as potential biomarkers for the proliferative stage of diabetic retinopathy.

Another study explored the association between C-peptide (CP)-related parameters and diabetic retinopathy in T2DM [298]. Findings indicate that impaired β-cell function, rather than insulin resistance, is more closely linked with diabetic retinopathy. In particular, the postprandial C-peptide-to-glucose ratio may serve as a valuable systemic marker for identifying T2DM patients at high risk for developing diabetic retinopathy, especially vision-threatening forms.

Finally, a study was conducted to evaluate and compare the potential of several inflammatory markers, neutrophil-to-lymphocyte ratio (NLR), platelet-to-lymphocyte ratio, systemic inflammation index, and red blood cell distribution width (RDW), as predictors of diabetic retinopathy severity in a U.S. population [299]. Results indicate that RDW combined with NLR presents a promising approach for predicting diabetic retinopathy severity.

Metabolite- and lipid-derived biomarkers

There is a broad consensus that metabolic dysregulation plays a crucial role in the development of diabetic vascular complications. A comprehensive study identifies six metabolites, including creatinine, albumin, tyrosine, glutamine, lactate, and the ratio of phospholipids to total lipids in small LDL, as being correlated with macrovascular complications. Eight additional metabolites, including glucose, tyrosine, very large high-density lipoprotein particles, valine, free cholesterol to total lipids in very small very low-density lipoprotein, alanine, albumin, and isoleucine, are associated with microvascular complications [300]. These findings provide compelling evidence that circulating metabolites can be utilized as predictive biomarkers for vascular complications in diabetic patients.

Sphingomyelin (SM) appears to play a role in the early stages of diabetic retinopathy. Evidence shows that average plasma levels of total SM are significantly lower in individuals with T2DM compared to those with prediabetes and healthy controls [301]. Additionally, lower plasma SM levels are associated with reduced retinal sensitivity in diabetic individuals, suggesting that SMs could be promising biomarkers for detecting diabetic retinal neurodegeneration at early disease stages.

Another study highlights that dodecanoylcarnitine, linoleylcarnitine, stearylcarnitine, decanoic acid, and proline could potentially serve as independent biomarkers for predicting therapeutic outcomes following vitrectomy in patients with type 2 diabetic retinopathy [302].

The visceral adiposity index, lipid accumulation product, and atherogenic index of plasma are established biomarkers for predicting metabolic disorders such as diabetes mellitus and associated microvascular complications, particularly diabetic kidney disease (DKD) and diabetic retinopathy. While a study shows these indices are significant predictors of DKD in individuals with diabetes mellitus, their utility in detecting diabetic retinopathy is limited [303].

Thickness changes in the outer plexiform layer may correlate with renal-related diseases such as diabetes

Given the similar pathophysiology of microvascular complications in diabetic kidney disease and diabetic retinopathy, a study was conducted to assess the relationship between outer plexiform layer (OPL) thickness and kidney function indicators such as estimated glomerular filtration rate (eGFR) and urine albumin-to-creatinine ratio (UACR) [304]. Perifoveal OPL thickness was found to be inversely associated with eGFR and positively associated with UACR. The authors suggest that OPL thickening could serve as a potential indicator for diabetic kidney disease, with optical coherence tomography (OCT) imaging offering a noninvasive means of exploring retinal–renal interactions.

#### 6.1.2. Nephropathy

Diabetic nephropathy is the most frequent complication of T2DM that affects kidney function, leading to a gradual decline in the renal capacity to filter waste products from the bloodstream [305]. It contributes to the development of end-stage renal disease (ESRD) [306], the final stage of chronic kidney disease, which results in permanent renal failure, underscores its significant clinical importance. While the pathogenesis of diabetic nephropathy is not fully understood, its development involves multiple factors, including metabolic, hemodynamic, growth factors, and proinflammatory and profibrotic pathways, all of which play significant roles [307,308,309]. Over time, persistent hyperglycemia can lead to deterioration of various kidney compartments, including the glomeruli, tubules, interstitium, and vasculature [310]. Besides hypertension and certain immune disorders, elevated blood glucose levels can cause microalbuminuria, characterized by the presence of small amounts of albumin in the urine. This condition leads to glomerular hyperfiltration and overt proteinuria, the hallmark of diabetic kidney disease and a risk factor for cardiovascular disease.

##### Potential Biomarker Options for Nephropathy

Dysregulated miRNA in diabetic kidney disease

Numerous miRNAs have been identified as either risk or protective factors in diabetes-related complications [311]. One study revealed a significant positive association between serum hsa-miR-221 and fasting insulin, fasting glucose, homeostatic model of assessment of insulin (HOMA-IR), albumin-to-creatinine ratio (ACR), and body mass index (BMI), with high specificity and sensitivity in patients with diabetic nephropathy [312]. Additionally, the expression levels of microRNA (miR)-132, miR-133a and long non-coding RNA megacluster (lnc-MGC), and their correlations with lactate dehydrogenase (LDH) and HbA1C, have been shown to serve as biomarkers distinguishing diabetic patients with reduced cardiovascular disease risk from those in early-stage diabetes [313]. Homo sapiens (has)-miR-221, in particular, appears to be a promising prognostic and diagnostic biomarker in diabetic nephropathy.

Growth Factors

Circulating levels of vascular endothelial growth factor (VEGF) family members are often elevated in individuals with T2DM. A study examining placental growth factor (PlGF), soluble fms-like tyrosine kinase-1 (sFLT-1), and VEGF-A highlighted their involvement in cardiorenal complications in patients with T2DM [314]. The study found elevated PlGF levels, as well as increased sFLT-1 and PlGF/sFLT-1 ratios, to be useful indicators of cardiorenal events in T2DM and diabetic kidney disease. However, treatment with canagliflozin did not reduce these biomarkers.

Additionally, elevated circulating levels of adipocyte fatty acid-binding protein (AFABP), fibroblast growth factor 21, and pigment epithelium-derived factor are positively correlated with markers of metabolic syndrome and microvascular complications, including the progression of nephropathy in Chinese patients with T2DM [315,316,317,318]. Notably, serum AFABP has shown stronger predictive value for incident sight-threatening diabetic retinopathy than for nephropathy.

Biomarkers of oxidative stress and inflammation

Circulating biomarkers such as IL-6, IL-10, CD163, CXCL9, CCL22, GDF15, IL-33, FGF21, follistatin, and neurofilament light chain (NfL) have been associated with both microvascular and macrovascular complications, including neuropathy, nephropathy, retinopathy, and major adverse cardiovascular events (MACE) [319]. Among these, CXCL9, GDF15, NfL, and FGF21 have been identified as independent predictors of mortality in T2DM.

A targeted proteomics approach compared inflammatory profiles between individuals with T1DM, T2DM, and healthy controls [320]. The results revealed that inflammatory proteins linked to nephropathy were similar across both types of diabetes. Specifically, fms-related tyrosine kinase 3 ligand (FLT3L) and extracellular newly (EN) identified receptors for advanced glycation end-products (RAGE) binding protein (EN-RAGE) were associated with cardiovascular disease in T2DM. While both T1DM and T2DM showed elevated levels of inflammatory proteins, the increase was more pronounced in T2DM.

Circulating activin A, an inflammatory mediator implicated in profibrotic kidney injury, is elevated in diabetic kidney disease and correlates with kidney damage. Animal studies suggest that activin A promotes kidney injury through macrophage-driven inflammation [321]. Inhibitors targeting activin A reduce senescence markers (e.g., p19), proinflammatory and pro-fibrotic markers, improve kidney morphology, restore podocyte markers (nephrin and Wilms tumor-1), and reduce albuminuria and fibrosis.

Growth differentiation factor 15 (GDF-15), a homeostatic cytokine, also plays a protective role in diabetic nephropathy [322]. Its anti-inflammatory actions and upregulation of renal–protective pathways suggest that GDF-15 may serve as both a diagnostic and prognostic biomarker.

Elevated levels of serum adhesion molecule-like protein, coupled with reduced levels of nesfatin-1 and 25-hydroxy vitamin D (25(OH)D), have been linked to a higher risk of diabetic kidney disease in T2DM patients [323].

Oxidative stress is a key factor in the development and progression of T2DM. Peroxiredoxin-4, an antioxidant protein, was found to be associated with increased risk of nephropathy independent of low-grade inflammation, but not with new-onset retinopathy or neuropathy [324].

Ischemia-modified albumin is a novel marker of oxidative stress. Elevated levels have been detected in patients with T2DM and are associated with the severity of diabetic complications such as retinopathy, nephropathy, and peripheral arterial disease [325]. Despite its low specificity, this assay may still be useful for risk assessment.

The oxidative stress-related metabolite 8-hydroxy-2′-deoxyguanosine (8-OHdG) has also been evaluated in diabetic patients with and without renal complications. Serum 8-OHdG levels were significantly higher in those with diabetic kidney disease, supporting its potential as a biomarker for oxidative DNA damage in diabetes-related renal dysfunction [326].

Hepatic and cardiac biomarkers

Cardiac biomarkers such as N-terminal prohormone of B-type natriuretic peptide and troponin T have shown associations with the progression of diabetic nephropathy [327]. Elevated levels of these markers in diabetic patients indicate advancing kidney dysfunction.

Angiopoietin-like protein 8, a hepatic-derived protein, has also been identified as a risk factor for diabetic nephropathy. Its significant elevation in affected patients suggests its utility as a potential biomarker [321].

#### 6.1.3. Neuropathy

Diabetic neuropathy, a debilitating complication of T2DM, encompasses a broad range of clinical pathologies manifested through a set of nerve disorders caused by nerve fiber damage due to abnormally high levels of blood glucose. Prolonged hyperglycemia can induce neuronal injury through several biochemical pathways, including oxidative stress, polyol pathway alteration, protein kinase C activation, and advanced glycation end-products formation [328]. There is a link between the severity of diabetes and both peripheral somatic and central neurodegeneration [329,330]. Diabetic neuropathy in T2DM is associated with dyslipidemia, central obesity, hypertension, insulin resistance, hormonal imbalance, and poor glucose control. It can affect multiple components of the nervous system, from the cerebral cortex to skeletal muscle, leading to a broad range of symptoms.

Diabetic neuropathy refers to various conditions involving damage to the autonomic or peripheral nervous systems. It involves both tissues (large and small vessels) and fibers (large and small nerve fibers) [331]. Autonomic neuropathy is a condition that impairs the normal functioning of the autonomic nervous system, leading to cardiovascular morbidity and mortality, with a clinical course that damages the nerves of the cardiovascular system, digestive system, thermoregulation, kidneys, and bladder [332,333]. While its causes are multifactorial, prolonged elevation in blood glucose levels can damage nerves over time. Common symptoms of this condition may include dizziness, an irregular heart rate, gastrointestinal disturbances, and bladder dysfunction.

Diabetic peripheral neuropathy, a form of peripheral nerve dysfunction, involves multiple types of nerve fibers and is classified into three subclasses according to fiber diameter: small-fiber, large-fiber, and mixed-fiber neuropathies [334]. It is associated with neuromuscular dysfunction and primarily affects large fibers or a combination of small and large fibers. The condition may result not only in skeletal muscular dysfunction but also in morphological alterations in the plantar tissue [335]. It reflects the complex interplay of immune, inflammatory, and vascular mechanisms. Due to its complexity, there is currently no effective treatment available, apart from maintaining a healthy lifestyle and tight control of blood glucose levels. There is a need to identify biomarkers that reflect the progression of the disease and improve therapeutic strategies.

##### Potential Biomarker Options in Neuropathies

Neuroinflammatory mediators

Chemokines play a key role in the pathogenesis of various neuropathies and neuropathic pain processes. Plasma levels of CXCL9, CXCL10, and CXCL11 have been measured in patients with neuropathy [336]. Among these, CXCL10 levels are significantly elevated in T2DM patients with neuropathy. It is suggested that CXCL10 may serve as an early detection biomarker, potentially aiding the development of therapeutic strategies to reverse or prevent diabetic neuropathy.

Netrin-1, a neurotrophic factor, has also been studied in relation to early diabetic neuropathy in patients with T2DM [337]. Serum netrin-1 levels show a gradual decline corresponding to the severity of small nerve fiber damage. This suggests that netrin-1 may serve as a biomarker for small fiber neuropathy in diabetes.

Glial Fibrillary Acidic Protein (GFAP), expressed in non-myelinating Schwann cells in the peripheral nervous system, and Ubiquitin C-terminal hydrolase L1 (UCH-L1), a neuron-expressed stress protein, are also of interest [338,339]. A study evaluating circulating GFAP and UCH-L1 levels in patients with and without diabetic polyneuropathy (DPN) found that serum GFAP levels were significantly reduced in individuals with DPN compared to controls and those without DPN [340]. This suggests that lower GFAP may indicate small nerve fiber damage, positioning GFAP as a potential biomarker for small fiber neuropathy.

Advanced glycation end products (AGEs) and their interaction with receptors for AGE (RAGE) are central to the pathogenesis of diabetic foot (DF), particularly in patients with neuropathy [341,342]. A study examined asymmetric dimethylarginine (ADMA), fructosamine, nitric oxide (NO), and soluble RAGE (sRAGE) in diabetic patients with and without neuropathy. Circulating sRAGE levels were significantly elevated in T2DM patients without DF compared to healthy controls. In contrast, ADMA and fructosamine levels were significantly higher in patients with DF, and NO levels were lower in this group compared to both non-DF diabetics and healthy individuals. The study suggests that sRAGE may act as an endogenous protective factor against the development of DF, with reduced levels potentially contributing to diabetic foot complications associated with neuropathy.

Hyperglycemia-induced molecules affecting metabolic and hemodynamic pathways

Heat shock protein 27 (Hsp27) is a small heat shock protein known for its role in protecting cells from apoptosis under stress. A study evaluated plasma Hsp27 levels in T2DM patients with and without microvascular complications such as diabetic retinopathy, diabetic nephropathy, and diabetic neuropathy [343]. Notably, Hsp27 levels were highest in the diabetic nephropathy group compared to controls and other complication groups, suggesting that plasma Hsp27 may serve as a potential biomarker for diabetic nephropathy.

Persistent hyperglycemia also alters hemoglobin (Hb) and red blood cell deformability, impairing hemorheology in T2DM [344]. Glycated hemoglobin reflects prolonged hyperglycemia. Another study found evidence of RBC breakdown and low-grade intravascular hemolysis (IVH) in T2DM patients [345]. Increased heme-related absorbance was associated with peripheral sensory neuropathy, but not with other vascular complications. These findings support an association between T2DM and low-grade IVH, which may contribute to the development of diabetic neuropathy.

### 6.2. Macrovascular Complications

#### 6.2.1. Coronary Artery Disease

Coronary artery disease (CAD), a macrovascular complication, occurs when plaque buildup (atherosclerosis) in the arteries of the heart restricts blood flow. Atherosclerosis is a progressive condition that affects multiple arteries throughout the body. CAD is associated with diabetes, hypertension, metabolic disturbances, lifestyle factors, and older age [346].

Individuals with T2DM may develop cardiovascular complications such as CAD, cardiac autonomic neuropathy, or diabetic cardiomyopathy [5]. Risk factors unique to diabetes increase the likelihood of CAD, contributing to atherosclerotic plaque formation and thrombosis [347,348,349]. Diabetes-accelerated CAD is complex and involves numerous metabolic and molecular signaling pathways, including hyperglycemia, oxidative stress, chronic inflammation, and epigenetic dysfunction within the coronary arteries [350].

Atherosclerosis-induced reduction in blood flow to the heart results in angina. It encompasses a broad range of conditions, including stable angina, unstable angina, myocardial infarction (MI), and sudden death [351,352,353]. The Framingham study indicates that diabetic patients are more likely to experience MI than those without diabetes [354]. Diabetic individuals without a prior history of MI are still at high risk of myocardial ischemia, heart attacks, and strokes [355]. Thus, T2DM-induced atherosclerosis is a significant global health concern.

##### Potential Biomarkers in Coronary Arterial Disease

Hormones as biomarkers

There is a strong association between the early stages of chronic kidney disease (CKD), vascular remodeling, and coronary artery calcification. Adropin, a nutritionally regulated peptide hormone primarily synthesized in the liver, is also produced in other tissues such as the brain, heart, and gastrointestinal tract [356]. Adropin plays a regulatory role in several cardiovascular pathologies [357]. A study has shown an association between adropin levels and asymptomatic coronary calcification in patients in the early stages of CKD [358]. Patients with known asymptomatic coronary artery calcification exhibited significantly reduced levels of circulating adropin. This suggests that low adropin levels may serve as a potential biomarker for predicting the risk of coronary artery calcification in early-stage CKD patients.

Endothelin-1 (ET-1), an endogenous vasoconstrictor, also plays a significant role in coronary artery disease (CAD) and diabetes. Its prognostic value has been evaluated in patients with stable CAD across different states of glucose metabolism [359]. Elevated ET-1 levels are significantly associated with an increased risk of cardiovascular events, suggesting that ET-1 could serve as a potential predictor in CAD patients with impaired glucose metabolism.

Oxidative stress

Endothelial dysfunction and oxidative stress are key contributors to the initiation and progression of atherosclerosis. A study investigated their roles in patients with severe CAD undergoing coronary artery bypass graft (CABG) surgery, comparing those with and without T2DM [360]. Although serum levels of superoxide dismutase 1 (SOD-1) and lectin-like oxidized low-density lipoprotein receptor-1 (LOX-1) were not affected by the presence of T2DM, diabetic patients exhibited significant endothelial dysfunction, as evidenced by impaired brachial flow-mediated dilation and altered serum serotonin (5-HT) levels. The study found that circulating 5-HT levels were markedly influenced by T2DM and could potentially serve as a biomarker for CAD severity.

Ischemia with non-obstructive coronary arteries (INOCA) is a frequent cause of hospital admissions [361], with diabetes-induced coronary microvascular dysfunction playing a role in its complications. MicroRNAs (miRNAs) are emerging biomarkers for endothelial dysfunction and cardiovascular diseases. One study examined circulating miRNAs involved in endothelial regulation in INOCA patients with and without diabetes [362]. It found that miR-363-5p and miR-92a-3p were significantly dysregulated in INOCA patients with diabetes, suggesting their potential as biomarkers for monitoring and predicting endothelial dysfunction in this group.

Another study highlighted an association between circulating calprotectin, a protein secreted by activated monocytes and neutrophils, and the development of ASCVD, defined by events such as myocardial infarction, stroke, coronary revascularization, or cardiovascular death [363,364]. Calprotectin, involved in inflammatory processes, was associated with several risk factors, including elevated hemoglobin A1c, triglycerides, very low-density lipoprotein (VLDL) cholesterol, and reduced HDL cholesterol and cholesterol efflux capacity. The findings suggest that calprotectin may serve as a mechanistically relevant biomarker for ASCVD, independent of conventional cardiovascular risk factors.

Metabolic messengers

Dyslipidemia is a well-established risk factor for ASCVD, particularly in T2DM patients. One study explored the association between dyslipidemia, dysglycemia, and subclinical coronary atherosclerosis [365]. It found that HDL diameter, free cholesterol, phospholipids, and total lipids in extra-large HDL were inversely associated with the coronary artery calcium score. These findings indicate that alterations in HDL composition and concentration are linked to both dysglycemia and subclinical coronary atherosclerosis.

Sphingosine 1-phosphate (S1P) is a lipid signaling molecule with potent immunomodulatory and anti-inflammatory effects. A study revealed that altered circulating levels of S1P and its precursors are associated with increased cardiovascular risk in T2DM [366], suggesting their potential use as biomarkers for coronary atherosclerosis.

Another study investigated the oxLDL/LDL-C ratio and its relationship with the severity of coronary atherosclerosis in T2DM patients [367]. Results indicated that a higher oxLDL/LDL-C ratio was positively associated with more severe coronary atherosclerosis, supporting its potential use as a biomarker for early detection and intervention.

Indicators of cell damage

Small leucine-rich proteoglycans (SLRPs), a type of matrix protein, have been associated with atherosclerotic plaque development [368]. One study examined the relationship between circulating lumican, a proteoglycan member of SLRPs, levels and CAD severity [369]. Serum lumican levels were elevated in patients with advanced CAD and demonstrated moderate predictive value (sensitivity: 64%, specificity: 65%) for disease severity.

Diabetes is a known risk factor for both CAD and chronic heart failure. Endothelial progenitor cells (EPCs), derived from bone marrow, play a critical role in vascular repair following injury. A study examined the response of circulating EPCs (cEPCs) to empagliflozin, an SGLT2 inhibitor, in diabetic patients with stable CAD [370]. Treatment resulted in increased cEPC levels and enhanced function, suggesting that EPC levels could serve as an indicator of therapeutic effectiveness.

A separate study evaluated the effects of empagliflozin versus placebo on high-sensitivity cardiac troponin-I (hs-cTnI) and lipid profile markers [371]. While the drug had a modest effect on lipid biomarkers, it significantly reduced circulating hs-cTnI levels, indicating improved cardiomyocyte function in T2DM patients with CAD.

#### 6.2.2. Cerebrovascular Disease

T2DM increases the risk of cerebrovascular disease, a macrovascular complication. The progression of diabetes perturbs metabolic pathways [372], intracellular lipids [373], and blood glucose levels [374], leading to structural and functional alterations in the endothelial and smooth muscle cells of cerebral blood vessels. Moreover, insulin resistance combined with persistent hyperglycemia and lipotoxicity triggers macrophage-derived foam cell formation [375,376,377]. Diabetes-induced macrovascular dysfunction promotes atherosclerosis, leading to luminal narrowing [378]. Ultimately, diabetic atherosclerosis contributes to ischemic processes when a cerebral blood vessel becomes blocked. Ischemic stroke can result from an embolus originating in the heart or a more proximal artery, which then obstructs a cerebral artery already affected by an atherosclerotic plaque, increasing stroke risk.

A second type of stroke is a hemorrhagic stroke. While hemorrhagic strokes have been observed in individuals with diabetes [379], their occurrence is associated with abnormally high HbA1c levels (≥72 mmol/mol). Poorly controlled blood glucose appears to significantly contribute to the risk of hemorrhagic stroke. These findings suggest that one of the primary vascular changes in T2DM is the development of atherosclerosis, which can lead to serious health complications.

##### Potential Biomarkers of Cerebrovascular Disease

Biochemical indicators

Subclinical atherosclerosis begins to develop years before cardiovascular and cerebrovascular diseases become symptomatic. A study examined the effects of subclinical atherosclerosis on carotid intima–media thickness (CIMT) in patients with prediabetes and T2DM [380]. The results showed a significant elevation in circulating oxidized LDL (oxLDL), soluble lectin-like oxidized LDL receptor-1 (sLOX-1), and proprotein convertase subtilisin/kexin type 9 (PCSK9) levels in T2DM patients with macrovascular complications compared to controls, prediabetes, and uncomplicated diabetes groups. Concentrations of oxLDL, sLOX-1, and PCSK9 were significantly associated with CIMT. These findings suggest that these biomarkers may serve as potential indicators for cardiovascular risk assessment in patients with prediabetes and T2DM.

Chitotriosidase, a glycosyl hydrolase, is primarily synthesized by macrophages and epithelial cells [381]. It is secreted in response to local microenvironmental cues and plays a role in immune-driven processes, including inflammation. Studies show that serum chitotriosidase levels are associated with the severity of atherosclerotic lesions in patients with atherothrombotic stroke and ischemic heart disease [382], suggesting its potential as a marker for atherosclerotic burden.

Chronic kidney disease (CKD) is an independent risk factor for MACEs and negatively impacts cerebrovascular function. Given the association between CKD progression and increased sympathetic tone, research has explored the relationship between cerebrovascular risk and renalase (RNL), a catecholamine-metabolizing enzyme, in pre-dialysis CKD patients [383]. Serum RNL levels were strongly associated with estimated glomerular filtration rate (eGFR), especially in relation to CKD progression. These findings suggest that RNL may serve as a predictor of renal and cerebrovascular outcomes.

Klotho is involved in the regulation of mineral metabolism, including phosphate and calcium. Mutations in the klotho gene have been linked to hypertension and kidney disease. Evidence indicates that klotho suppresses oxidative stress [384], improves endothelial function, and provides vascular protection [385]. Studies investigating its role in diabetic vascular complications found that elevated klotho levels are associated with a reduced risk of coronary artery disease and cerebrovascular events [385]. Notably, klotho levels independently predicted the development of macroangiopathies over a seven-year period. Circulating klotho is thus considered a valuable predictor of long-term macrovascular outcomes in T2DM patients.

Neovasculogenesis

A separate study investigated the number and function of circulating endothelial progenitor cells (EPCs) in various vascular complications of T2DM and their association with vascular endothelial function [386]. The study found a reduction in both the number and function of circulating EPCs in T2DM patients, with the degree of reduction varying across different vascular diseases. EPC counts were correlated with endothelial function, suggesting that circulating EPCs may be used as a surrogate biomarker for vascular endothelial function in T2DM.

Moyamoya disease (MMD) is a chronic cerebrovascular disorder characterized by progressive occlusion of the internal carotid arteries or their branches [387]. A study explored the presence of EPCs and circulating endothelial cells (CECs) in patients with MMD [388]. CEC counts were significantly elevated in patients compared to controls. EPC counts were independently associated with patient age, while CEC counts were negatively associated with comorbid conditions such as hypertension, diabetes mellitus, and coronary heart disease.

#### 6.2.3. Peripheral Artery Disease

PAD, a macrovascular complication, is characterized by the narrowing of arteries due to plaque buildup. As a chronic condition, PAD can lead to partial or total peripheral vascular occlusion. It involves atherosclerosis of the lower extremity arteries, the severity of which depends on the angiogenic response that triggers the development of collateral circulation to reduce ischemia and improve outcomes. However, functional impairment may increase the risk of atherothrombosis—the formation of a blood clot at the site of an atherosclerotic plaque.

Diabetes significantly contributes to PAD through various mechanisms, including glycation, inflammation, lipid abnormalities, insulin resistance, and endothelial dysfunction [389,390]. There is a significant association between PAD and male gender, former smoking, cerebrovascular disease, age, duration of diabetes, and high-density lipoprotein (HDL) levels [391].

PAD may cause multiple symptoms. In individuals with type 2 diabetes mellitus (T2DM), intermittent claudication may occur via nociceptive, inflammatory, and neuropathic mechanisms [392,393,394]. Intermittent claudication is classified into two subtypes: intermediate neurogenic claudication and intermediate vascular claudication [395]. Diabetic patients with PAD may present with variable intermittent claudication. Evidence suggests that some individuals with diabetes may not exhibit typical symptoms, as vascular claudication can be masked by peripheral neuropathy [391], while others may present with claudication and arterial ulceration. Notably, an association has been observed between plasma fibrinogen levels and diabetic foot ulcers, along with various clinical and inflammatory biomarkers [396].

Other symptoms include cold extremities due to poor circulation, reflecting reduced arterial blood flow, along with the development of non-healing ulcers or sores on the lower limbs [397,398]. Potential complications of PAD in individuals with diabetes include heart attack and stroke, which are among the leading causes of disability and death worldwide.

##### Potential Biomarker Options for Peripheral Arterial Disease

Blood-based factors

PAD is a common vascular condition. A study utilizing high-throughput proteomic profiling identified biomarkers associated with PAD [399]. Notably, plasma beta2-microglobulin (B2M) levels were significantly higher in PAD patients compared to non-PAD patients with coronary artery disease. B2M levels correlated with disease severity independently of other risk factors, as well as with the ankle–brachial index and functional capacity. These findings suggest B2M may serve as a promising biomarker for PAD.

Another study evaluated the predictive potential of circulating endothelium-enriched microRNA-126 (miR-126) in T2DM patients, both with and without coronary artery disease (CAD) [400]. miR-126 showed a strong association with both T2DM and CAD.

Endostatin, a cleavage fragment of collagen XVIII, is known to inhibit angiogenesis [401]. A study investigated the relationship between circulating endostatin levels and lower limb PAD symptoms [402]. Results showed elevated serum endostatin in older men experiencing intermittent claudication, supporting its role as a potential PAD biomarker.

Inflammatory mediators

The relationship between vascular indices and circulating inflammatory biomarkers was explored in T2DM patients with poor glycemic control and no known cardiovascular disease [403]. Chitinase-3-like protein 1 (YKL-40) and neutrophil gelatinase-associated lipocalin (NGAL) emerged as novel indicators of vascular inflammation, showing associations with subclinical atherosclerosis in this population.

Adiponectin, a protein primarily secreted by adipocytes, circulates in peripheral blood [404]. Low levels of adiponectin (<4 µg/mL), or hypoadiponectinemia, are linked to several diseases, including coronary artery disease, stroke, and PAD. Adiponectin is therefore considered a biomarker of atherosclerosis.

Soluble urokinase plasminogen activator receptor (suPAR) is a marker of immune activation and is associated with atherosclerosis. A study assessed the relationship between suPAR and prevalent PAD [405], finding that elevated plasma suPAR levels predicted both existing PAD and future cardiovascular and PAD-related events.

Cell-derived molecules

Patients with PAD are at increased risk for MACE. Insulin-like growth factor-binding protein 1 (IGFBP-1), mainly produced in the liver, was found to be significantly elevated in patients who experienced MACE [406]. IGFBP-1 emerged as the only biomarker independently associated with MACE over a two-year follow-up in both male and female PAD patients.

Adipocyte fatty acid-binding protein (FABP4), a fatty acid chaperone secreted by adipocytes and macrophages, is elevated in T2DM. It contributes to lipolysis and acts as an adipokine involved in insulin resistance and atherosclerosis [407]. A study investigating circulating FABP4 levels and endothelial function in T2DM patients found a negative association, suggesting that elevated FABP4 may directly impair vascular endothelium and serve as a valuable marker of vascular integrity [408]. Additionally, circulating miR-126 has been proposed as a biomarker for predicting T2DM patients with diabetic CAD.

A separate study evaluated biomarkers predictive of MACE in PAD patients [409]. While matrix metalloproteinase-10 (MMP-10) was associated with MACE in diabetic individuals, matrix metalloproteinase-7 (MMP-7) was independently associated with 2-year MACE prognosis in PAD patients.

## 7. Conclusions

T2DM is a complex chronic disorder and a major risk factor for neuropathy, nephropathy, retinopathy, coronary artery disease, cerebrovascular disease, and peripheral vascular disease. The concept of personalized medicine is emerging as a transformative approach to tailor medical treatment to the specific needs and characteristics of each patient. Since the treatment of T2DM is primarily based on HbA1c levels, improved glycemic control, and the reduction in long-term complications, circulating predictive biomarkers may prove useful in assessing patient responses to specific treatments, helping to ensure optimal therapeutic benefit with minimal side effects.

**Figure 6:**
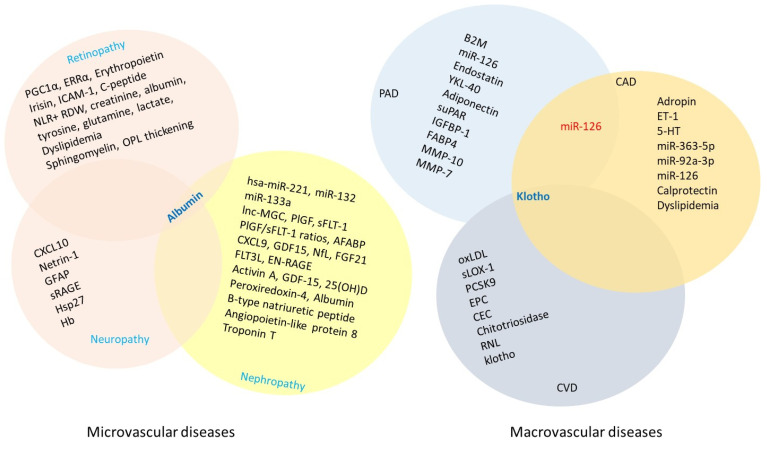
The emerging role of biochemical indicators in diabetic microvascular and macrovascular dysfunction. A variety of risk factors promote the development and progression of diabetic vasculopathy. Dyslipidemia combined with endothelial injury is a precursor of atherosclerosis. The common indicator between CAD and PAD is shown in “Red”. The common indicators of microvascular or macrovascular disease are shown in “Dark Blue”. PAD: peripheral artery disease, CAD: coronary artery disease; CVD: cerebrovascular disease.

The image shown in Figure 6 illustrates the biomarkers discussed in this review and their potential roles in T2DM. A range of genetic and non-genetic biomarkers already have established roles in T2DM, though not all are suitable for routine clinical use due to various limitations. For instance, in monogenic diabetes, genetic testing can identify mutations that not only confirm a diagnosis of maturity-onset diabetes of the young (MODY) but also enable targeted therapy based on the patient’s genetic profile. For individuals with MODY1 or MODY3, low-dose sulfonylureas are considered first-line treatments. Similarly, circulating biomarkers may guide drug choices in broader T2DM populations and contribute to reducing morbidity and mortality. Notably, high-sensitivity C-reactive protein has been identified as a sensitive test to help distinguish MODY from T2DM.

## Figures and Tables

**Table 1:** Potential biomarkers predicting the serious side effects of approved glucose-lowering medications by organ system (Node).

System (Node)	Serious Side Effects	Glucose-Lowering Medication *	Biomarker Test	Ref.
Cardiovascular	Euglycemic diabetic ketoacidosis Increased risk of ventricular arrhythmia Increased risk of sudden cardiac death Possible atherosclerotic cardiovascular disease Congestive heart failure Edema	Empagliflozin Sitagliptin Glipizide Pioglitazone Sitagliptin ^†^	Comprehensive metabolic panel plus serum ketones Troponins and brain natriuretic peptides Adipokines, sortilin, klotho, and FGF23 Brain natriuretic peptide, N-terminal brain natriuretic pro-peptide, cardiac troponins, growth differentiation factor-15 Brain natriuretic peptide	[171,172,173] [174,175,176,177] [111,112] [178,179,180,181,182,183] [123,184]
CNS	Peripheral neuropathy Dementia Diabetic macular edema Diabetic retinopathy	Metformin Glipizide Pioglitazone Semaglutide	Myelin protein zero circulating mRNA, neuron-specific enolase No blood-based biomarkers are available Vascular endothelial growth factor, interleukins, matrix metalloproteinases, and tumor necrosis factor, Fibroblast Growth Factor Optical coherence tomography biomarkers such as the absence of external limiting membrane and the presence of disorganization of the retinal inner layers Tumor necrosis factor-α, monocyte chemoattractant protein-1, serum extracellular vesicles’ miRNA	[185,186,187] [188] [189,190,191] [192] [193,194]
Endocrine	Increased hypoglycemia risk Pancreatitis	Glipizide Repaglinide Sitagliptin Semaglutide Sitagliptin	C-peptide and islet autoantibodies Serum lipase	[115,195,196,197] [198,199,200,201,202]
Gastrointestinal	Hepatotoxicity Vitamin B_12_ malabsorption and/or deficiency Gastroparesis Cholelithiasis and cholecystitis Gastrointestinal adverse effects Increased LDL-C levels	Metformin Semaglutide Empagliflozin	Aspartate aminotransferase and alanine aminotransferase Routine screening total serum B_12_ concentration No blood-based biomarkers are available 3-Oxotetradecanoic acid and 12-Hydroxydodecanoic acid No blood-based biomarkers are available Lipoprotein (a), high-sensitivity C-reactive protein, IL-6	[203] [204,205] [206,207] [208,209] [210] [211,212,213,214]
Hematological	Anemia A high risk of erythrocytosis Higher risk of hematocrit	Metformin Empagliflozin	Hemoglobin concentration, hematocrit (microcytic: <83 fL), and increased cytokine due to chronic inflammation Screen for V617F variant of Janus kinase 2	[215,216] [217,218,219,220,221,222]
Immune system	Angioedema Anaphylaxis Stevens–Johnson syndrome	Semaglutide Sitagliptin Semaglutide Sitagliptin Sitagliptin ^†^	Complement 1 (C1)/C1-inhibitor complex, complement 4, cleaved high molecular weight kininogen Mast cell tryptase α-defensin, lipocalin-2, receptor-interacting protein 3 (RIP3)	[223,224,225,226] [227,228,229,230,231] [232,233,234,235,236,237]
Musculoskeletal	Moderate fracture risk Increased risk of amputation (including minor and major amputation)	Pioglitazone Empagliflozin ^†^	Osteocalcin, bone specific alkaline phosphatase, carboxyterminal propeptide of type 1 collagen, and aminoterminal propeptide of type 1 collagen pro-oxidant balance, neutrophils-to-lymphocyte ratio	[121,122] [238,239]
Renal	Lactic acidosis Dose-related fluid retention Acute kidney injury due to volume depletion Dehydration	Metformin Repaglinide Pioglitazone Semaglutide Empagliflozin Semaglutide	Increased blood lactate level, arterial blood gas, and basic metabolic panel Kidney injury molecule-1, urinary neutrophil gelatinase-associated lipocalin test Rise in serum creatinine, cystatin C, urinary IL-9, and chitinase 3-like protein 1 Urinary neutrophil gelatinase-associated lipocalin test, kidney injury molecule-1	[240,241,242,243,244] [123,245,246] [247,248] [246,249]

* Indicates that the drug may also be used for other conditions; ^†^ Denotes caution is warranted, as data regarding this medication have been revised and remain under investigation.

## Data Availability

The original contributions to the study are included in the article; further inquiries can be directed to the corresponding author.

## Abbreviations

The following abbreviations are used in this manuscript:

ACCORD	Action to Control Cardiovascular Risk in Diabetes
ADA	American Diabetes Association
CV	Cardiovascular
CNS	Central nervous system
CAD	Coronary artery disease
DCCT	Diabetes Control and Complications Trial
DPP-4	Dipeptidyl peptidase-4
FDA	Food and Drug Administration
GLP-1	Glucagon-like peptide-1
GIP	Glucose-dependent insulinotropic polypeptide
HbA1c	Plasma glycosylated hemoglobin A1c
HHS	Hyperosmolar hyperglycemic state
IDF	International Diabetes Federation
SGLT2	Sodium–glucose co-transporter-2
T1DM	Type 1 diabetes mellitus
T2DM	Type 2 diabetes mellitus
UKPDS	United Kingdom Prospective Diabetes Study
WHO	World Health Organization
